# Autocorrelation analysis of a phenotypic screen reveals hidden drug activity

**DOI:** 10.1038/s41598-024-60654-x

**Published:** 2024-05-02

**Authors:** Richard A. Dubach, J. Matthew Dubach

**Affiliations:** 1 Siren Pharmaceuticals, Boston, USA; 2 Institute for Innovation in Imaging, Massachusetts General Hospital, Harvard Medical School, Boston, USA

**Keywords:** Phenotypic screening, High-throughput screening, Fluorescence imaging

## Abstract

Phenotype based screening is a powerful tool to evaluate cellular drug response. Through high content fluorescence imaging of simple fluorescent labels and complex image analysis phenotypic measurements can identify subtle compound-induced cellular changes unique to compound mechanisms of action (MoA). Recently, a screen of 1008 compounds in three cell lines was reported where analysis detected changes in cellular phenotypes and accurately identified compound MoA for roughly half the compounds. However, we were surprised that DNA alkylating agents and other compounds known to induce or impact the DNA damage response produced no measured activity in cells with fluorescently labeled 53BP1—a canonical DNA damage marker. We hypothesized that phenotype analysis is not sensitive enough to detect small changes in 53BP1 distribution and analyzed the screen images with autocorrelation image analysis. We found that autocorrelation analysis, which quantifies fluorescently-labeled protein clustering, identified higher compound activity for compounds and MoAs known to impact the DNA damage response, suggesting altered 53BP1 recruitment to damaged DNA sites. We then performed experiments under more ideal imaging settings and found autocorrelation analysis to be a robust measure of changes to 53BP1 clustering in the DNA damage response. These results demonstrate the capacity of autocorrelation to detect otherwise undetectable compound activity and suggest that autocorrelation analysis of specific proteins could serve as a powerful screening tool.

## Introduction

Phenotypic screening is a potent tool to identify compounds that alter cellular function or properties^1^. Historically, phenotypic screening has played a significant role in identifying many of the drugs that are currently in the clinic^2^. More recent applications of phenotypic screening, or image based profiling^3^, employ high throughput fluorescence imaging of cells with fluorescent labels that capture the shape and structure of the cell and organelles^4^. Morphological profiling of large datasets^5^ enables assignment of compound mechanism of action by comparing properties to known compounds and can also incorporate the impact of genetic perturbations^6^. Because these screens don’t rely on a priori knowledge of key targets, they can provide profound insight in the drug discovery pathway^7^.

53BP1 is a component of the DNA double strand break response pathway and recruited to sites of DNA damage into foci that form around damaged DNA^8^. These foci are traditionally studied through visualization of fluorescently labeled 53BP1^9–11^. There are myriad cellular properties that impact 53BP1 recruitment and function, including ATM activity^12^, cell cycle^13^ and epigenetic modifications^14^. Therefore, compounds that induce DNA damage, alter the cell cycle, impact DNA signaling or alter the DNA damage response are expected to affect the recruitment of 53BP1 to DNA, impacting the distribution pattern within the nucleus. In theory, any altered localization of fluorescently labeled 53BP1 would induce a detectable phenotypic change to reveal compounds with activity that induce or impact DNA damage.

Recently phenotypic profiling of 1008 compounds enabled mechanism of action (MoA) identification of compounds with unclear mechanisms through comparison to known MoAs^15^. The profiling used up to 58 features to capture induced phenotypes in three different cells lines with 5 different fluorescent labels. Phenotypic analysis extracted features from segmented cells to classify compound MoA based on unique MoA descriptors. The screen accurately ranked roughly half the testable MoAs of the reference compounds. Yet, a substantial number of MoAs did not induce a phenotype. As an example, for all three cell lines, TP53BP1-CLTA labeled cells did not produce phenotypic screen sensitivity to compounds with known DNA damage, DNA damage response, or cell cycle MoAs. A surprising result considering 53BP1 is a canonical DNA damage response marker^16^.

Here we hypothesized that focused analysis of 53BP1 clustering would prove more sensitive as a screening tool than traditional phenotypic screening. We used image autocorrelation analysis of 53BP1 images to determine if 53BP1 localization within the nucleus is altered by compound treatment. Image autocorrelation enables quantification of the spatial heterogeneity of fluorophores that is not possible with traditional analysis due to background noise present in all fluorescent imaging^17^. Thus, autocorrelation provides a potentially more sensitive measurement of compound induced changes in 53BP1 localization. Indeed, we found image autocorrelation was broadly more sensitive than traditional phenotypic screening and capable of classifying alkylating agents by type where the phenotypic did not detect any activity.

## Results

**Figure 1:**
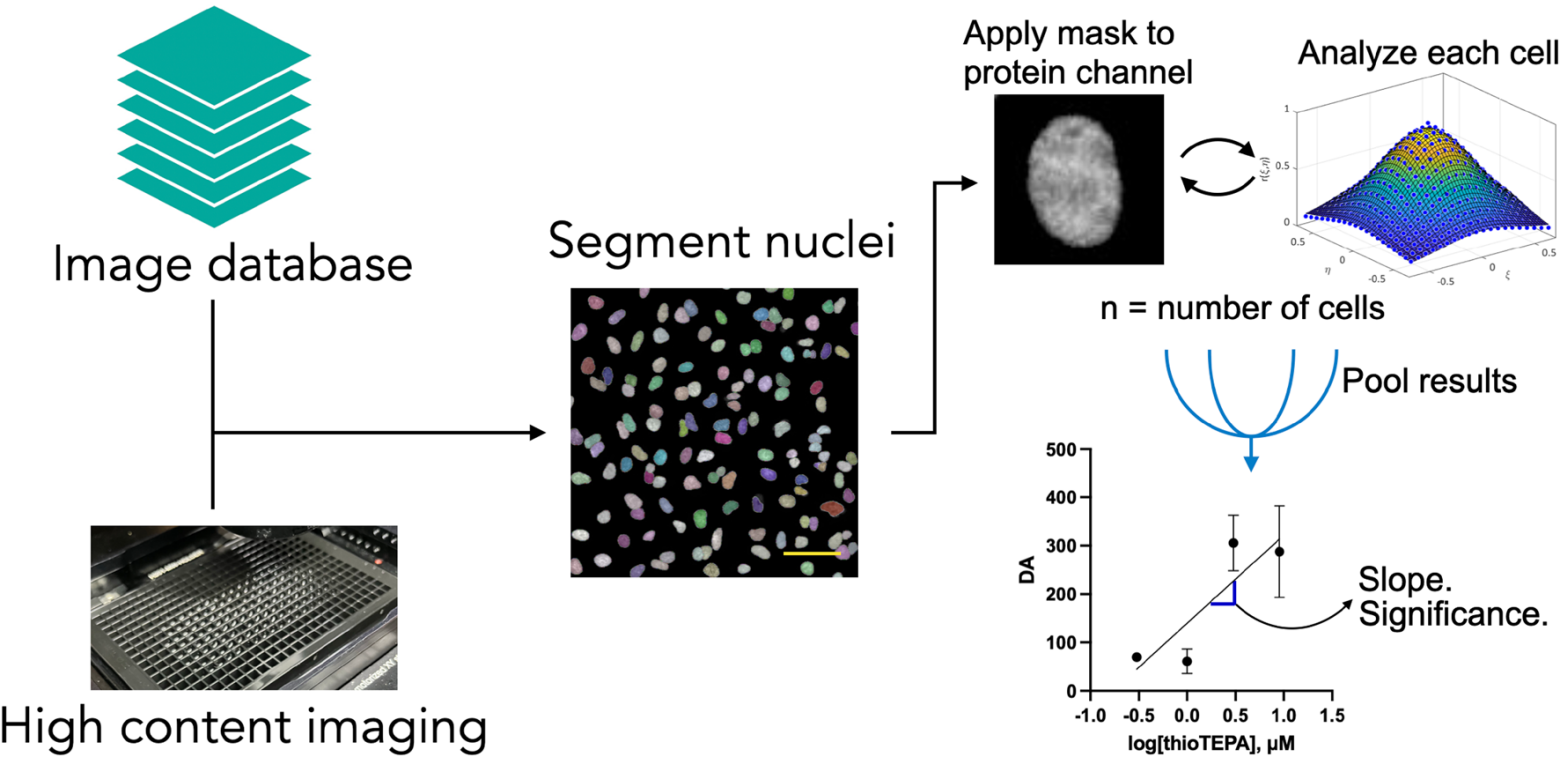
Autocorrelation analysis of screens or experiments. Images were accessed from the screen database or the microscope computer. Individual nuclei from each image where then segmented using Stardist on the BFP or DAPI channel. The nuclei mask was then applied the protein of interest channel and each cell was individually analyzed. Cells from all images at each condition were then pooled and the average and standard deviation were determined. A linear regression of autocorrelation DA versus compound concentration was then performed and the slope and significance versus the null hypothesis (slope = 0) were calculated to determine compound activity. Scale bar = 60 μm.

We accessed the original phenotypic screen^15^ images through the IDR API on the Open Microscopy server. The screen contained three different cell lines that had endogenous TP53BP1 labeled GFP, which generates GFP tagged 53BP1 protein. We first identified all the images from these three cell lines (> 60,000 images in total), then segmented cell nuclei in each image set using the BFP channel image (Fig. 1). Nuclei at the edge of images and false segmentations were removed by automatically removing segmented nuclei based on size. Segmented nuclear regions served as a mask for the TP53BP1 image and image autocorrelation was performed on each nucleus. The degree of aggregation (DA, a measurement of fluorophore clustering) was calculated over the entire nucleus and edge effects were removed by padding the segmented region with average intensity values for each nucleus. The DA for each nucleus was then averaged over all the cells in each condition to produce an overall image 53BP1 DA that corresponded to the compound concentration. The dataset contained repeats of four different doses for each compound. The activity of each compound in each cell line was then determined by fitting a linear regression to the average DA as a function of compound concentration. The slope and significance versus the null hypothesis (slope equal to zero) of DA versus concentration was then determined. Active compounds were defined as those having a significant (*p* < 0.05) slope of DA versus concentration.

53BP1 DA is a measure of protein labeled fluorophore clustering within the nucleus. A positive regression of DA versus concentration indicates that the compound induced 53BP1 recruitment to foci at sites of DNA repair and/or processing. Increases in 53BP1 recruitment can occur either through induction of DNA damage or altered repair pathways, such as shifting the response from homologous recombination to non-homologous end joining. Conversely, a negative regression indicates that the compound prevented 53BP1 recruitment to DNA damage or reduced the amount of DNA damage in the cell. However, it should be noted that, as a phenotypic measurement, there are other potential mechanisms of altered 53BP1 clustering that could drive observed compound activity. Yet, given the highly characterized role of 53BP1 in the DNA damage response^16^, recruitment to DNA damage response foci is likely the most prominent driver of measured activity.

**Figure 2:**
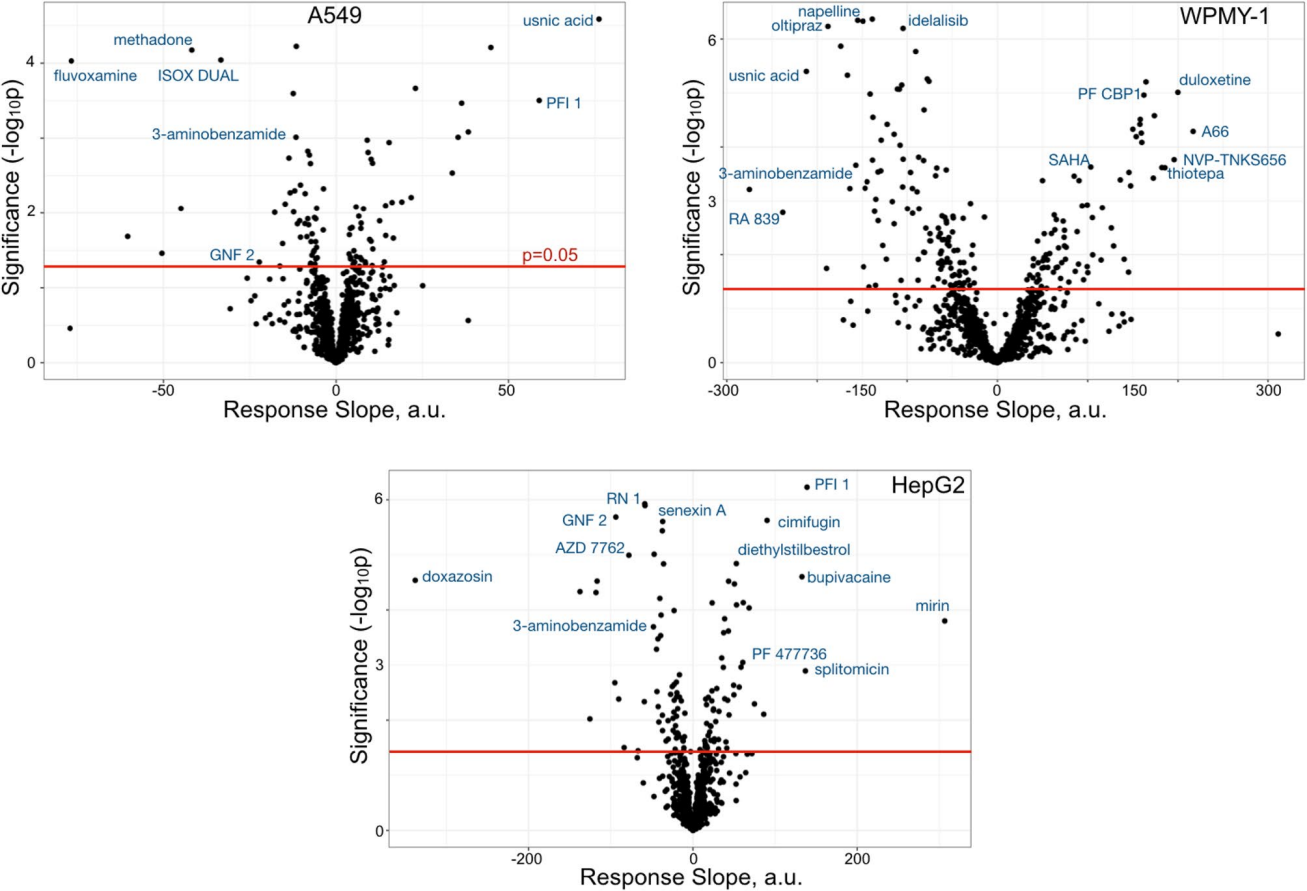
Compound activity on 53BP1 recruitment. Volcano plots for DA versus concentration response slope linear regressions for each cell line. Above the red line (*p* = 0.05) are compounds with significant activity. Identified compounds are known to impact the DNA damage response. Full results are in Table S1.

Volcano plots for each cell line were generated to visualize the results of autocorrelation analysis of the phenotypic screen (Fig. 2). A few compounds with significant activity and targets known to be involved in DNA handling, DNA damage response or cellular cycle were identified in the results. Table S1 contains the complete results. To best compare our results to those of the original screen we did not correct for multiple testing. The original screen did correct for MoA comparison but not for individual compound response. However, we did perform correction using Benjamini–Hochberg FDR adjustment of p-values and included those results in Table S1. This correction decreased the number of significant hits in the analysis. Overall, the majority of compounds with the strongest activity have mechanisms of action that impact the DNA damage response. Mirin, which inhibits Mre11 competition with 53BP1 at stalled replication forks^18^ produced a strong response in HepG2. Bromodomain inhibitors impact 53BP1 signaling^19^—PFI 1 showed strong positive activity in both HepG2 and A549 cell lines, while PF CBP1, another bromodomain inhibitor, had strong activity in WPMY-1 cells. Other compounds that impact the DNA damage response were also strong inducers of 53BP1 recruitment. These include, in WPMY-1 cells: A66 (a p110α selective PI3K inhibitor), thiotepa (a DNA alkylating agent), and SAHA (a HDAC inhibitor).

However, numerous compounds had significant activity reducing the recruitment of 53BP1. As an example, Nrf2 activators oltipraz and RA839 were two of the strongest 53BP1 recruitment-reducing compounds in WPMY-1 cells. Nrf2, a transcription factor, plays a role in the DNA damage response^20^ to promote homologous recombination repair^21^, which likely reduces 53BP1 recruitment. Other compounds that impact DNA damage also have activity in our analysis. For example, the ABL1 inhibitor GNF 2 reduced 53BP1 recruitment in both HepG2 and A549 cells, likely through decreasing DNA damage^22^. Curiously, usnic acid strongly induced 53BP1 recruitment in A549 cells but strongly prevented recruitment in WPMY-1 cells. However, the mechanism of action of usnic acid in the cellular DNA damage response remains unresolved^23^, warranting further exploration of the differences between these cell lines.

**Figure 3:**
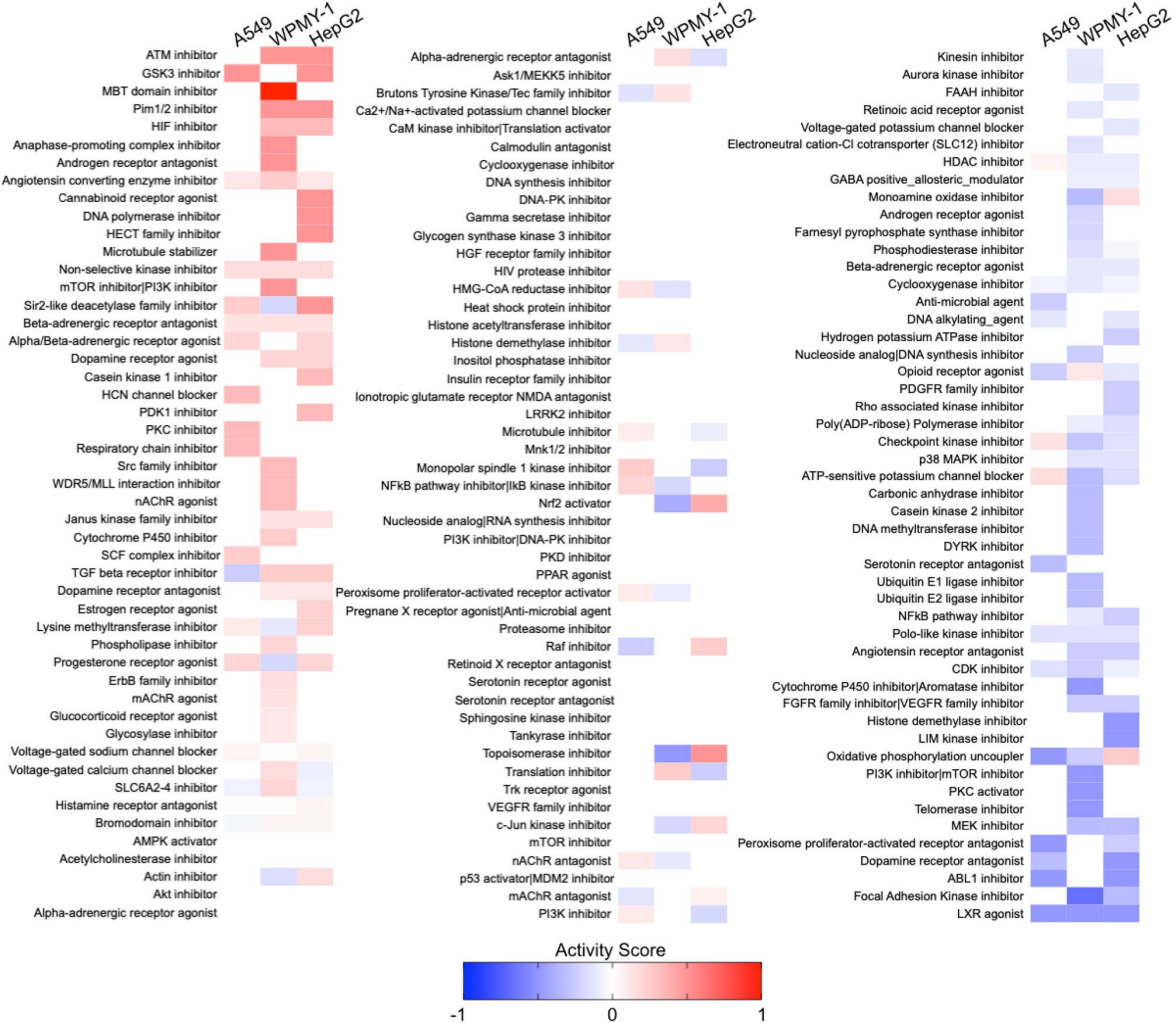
Mechanism of Action activity on 53BP1 clustering. Activity scores for mechanisms of action with at least 5 compounds.

We also determined a mechanism of action (MoA) activity score for each MoA containing results from at least 5 compounds through calculating the fraction of compounds with significant activity (Fig. 3). Here, we grouped compounds by the MoA defined in the original screen. Compounds that prevent 53BP1 recruitment have a negative score while compounds that increased 53BP1 clustering score positive. Here, we integrated the score of each compound within an MoA and divided by the number of compounds to generate an overall activity score between − 1 and 1. Thus, for MoAs with compounds that are both negative and positive, such as PARP inhibitors, the activity score is closer to zero than the total number of active compounds. The MoAs that had the most activity in inducing 53BP1 DNA damage recruitment are ATM, GSK3 and MBT domain inhibitors, all compounds that canonically impact the DNA damage response. Conversely, some of the lowest scoring MoAs were ABL1 inhibitors, which reduce DNA damage^22^, and FAK inhibitors and LXR agonists, which both impact DNA repair without clear roles^24,25^, mechanistically suggesting that 53BP1 recruitment is reduced in cells treated with these compounds.

Some MoAs contained compounds that both reduced or increased 53BP1 recruitment. For example, the PARP inhibitor 3-aminobenzamide reduced 53BP1 recruitment in each of the 3 cell lines. However, the PARP inhibitors NVP-TNKS656, NU 1025, and 4-HQN increased 53BP1 recruitment in at least one cell line. Given the history of PARP inhibitors being misclassified^26^ and the absence of more advanced clinical PARP inhibitors in the screen, these divergent results could stem from promiscuous or mis-identified compound MoAs.

In the original phenotypic analysis DNA alkylating agents had no measured activity—a surprising finding considering the role of 53BP1 signaling in the DNA damage response and response to alkylating agents^8,27^. However, our analysis found that DNA alkylating agents indeed have activity—33% in WPMY-1, 25% in HepG2, and 11% in A549 cells. Yet, the response was lower than expected, particularly in HepG2 and A549 cell lines. Unfortunately, images in the phenotypic screen have binned pixels. Binning serves to reduce the number of pixels over which the 53BP1 signal can be autocorrelated as well as increase the pixel size to limit spatial heterogeneity detection, which both impact the sensitivity of our analysis^28^. In the screen images, HepG2 cells had the smallest nuclei, while A549 cell nuclei were 15% larger and WPMY-1 cell nuclei were 40% larger. Therefore, autocorrelation analysis in WPMY-1 cells is expected to be more sensitive to changes in 53BP1 recruitment due to more pixels over which analysis can be performed, as we found. Thus, we hypothesized that the lack of analysis sensitivity to DNA alkylating agents was not a biological phenomenon, but rather a limitation from the screen measurements that used pixel binning during image acquisition. Therefore, we measured DNA alkylating agent impact on 53BP1 recruitment using non-binned pixels in the laboratory.

**Figure 4:**
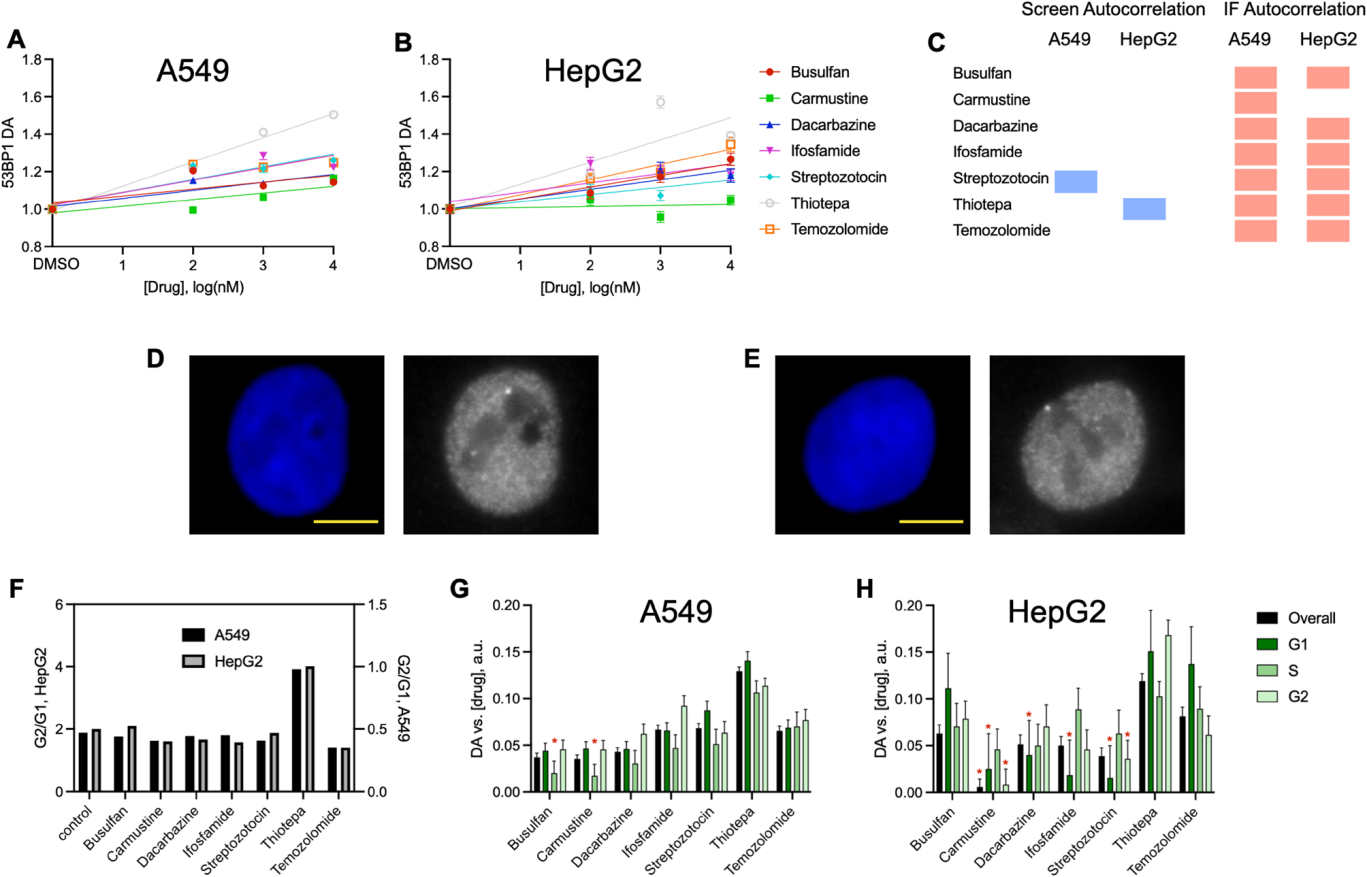
Experimental analysis of 53BP1 with non-binned pixels in imaging. (**A** and **B**) 53BP1 DA as a function of drug dose in A549 (A) and HepG2 (B) cells. Data are average with SEM, n > 1015 cells over 7 biological repeats for each condition, and linear regression. (**C**) Activity results of autocorrelation analysis response to alkylating agents. Shown are significant dose dependent reduction in clustering (blue), significant dose dependent increase in clustering (red) and no significance (no marker). (**D**) Representative DAPI (blue) and 53BP1 (white) images of an A549 cell treated with DMSO, scale bar 4 μm. (**E**) Representative DAPI (blue) and 53BP1 (white) images of an A549 cell treated with 1 μM Thiotepa, scale bar 4 μm. (**F**) Cell cycle classification G2/G1 ratio of all the cells analyzed following 1 μM treatment for 24 h of each compound. (**G** and **H**) Autocorrelation DA versus concentration linear regression slope for all cells (overall) and cells in each of the cell cycles for each of the alkylating agents in A549 (E) and Hep2 (F) cells. Shown are linear regression slope with standard error of the fit, red asterisks indicate responses that were not significantly different from zero (F number).

We focused on the smaller nuclei cells, A549 and HepG2 and selected 7 DNA alkylating agents that were used in the original screen. Cells were plated in 384 well plates, treated with compound for 24 h and imaged using immunofluorescence. Measurements of 53BP1 labeled through immunofluorescence generated a significant dose-dependent increase in 53BP1 clustering following treatment of all DNA alkylating agents in both cell lines except for carmustine in HepG2 (Fig. 4A–C). There was no resolvable difference in the distribution of 53BP1 between DMSO and treated cells. Examples of A549 cells treated with DMSO or 1 μM Thiotepa and imaged at 60x are shown in Fig. 4D,E. These results were in stark contrast to the sensitivity of autocorrelation analysis of the original dataset and more aligned with the expected response to DNA damaging agents. Thiotepa generated the strongest recruitment of 53BP1 to DNA damage. We then set out to determine how the cell cycle phase impacts 53BP1 signaling. Cells were classified by cycle based on their DAPI intensity, as previously described^29^. We found that thiotepa uniquely induced cells to stall in the G2 phase of the cell cycle (Fig. 4F). But, analysis of 53BP1 clustering by cell cycle demonstrated that the impact of each DNA alkylating agent was largely independent of cell cycle phase (Fig. 4G,H).

**Figure 5:**
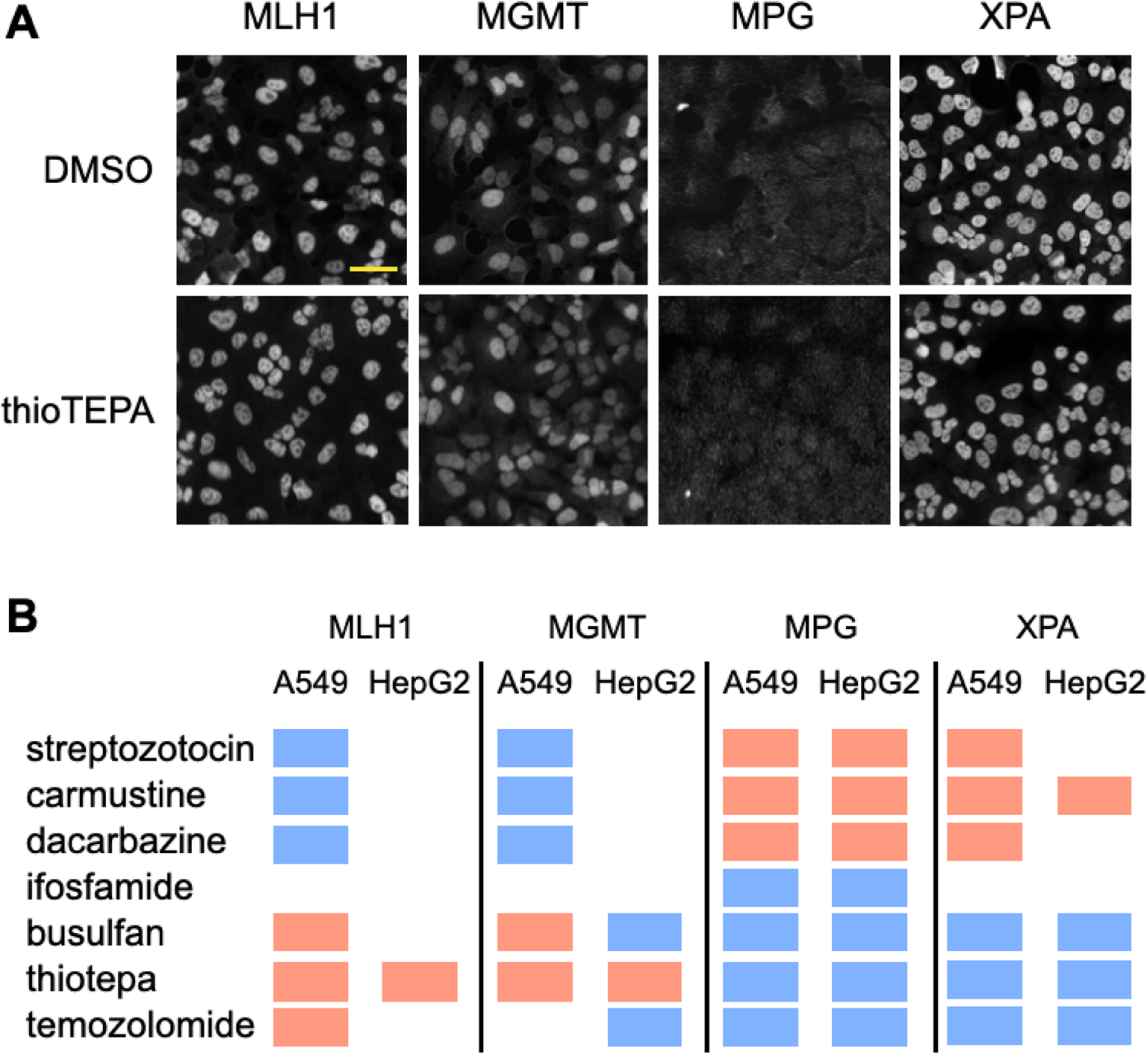
Autocorrelation analysis of DNA damage response proteins. (**A**) Representative immunofluorescence images of proteins in A549 cells. Cells were treated for 24 h with either DMSO or 1 μM thiotepa, scale bar = 40 μm. (**B**) Activity results of autocorrelation analysis for each protein in response to alkylating agents. Shown are significant dose dependent reduction in clustering (blue), significant dose dependent increase in clustering (red) and no significant response (no marker).

**Figure 6:**
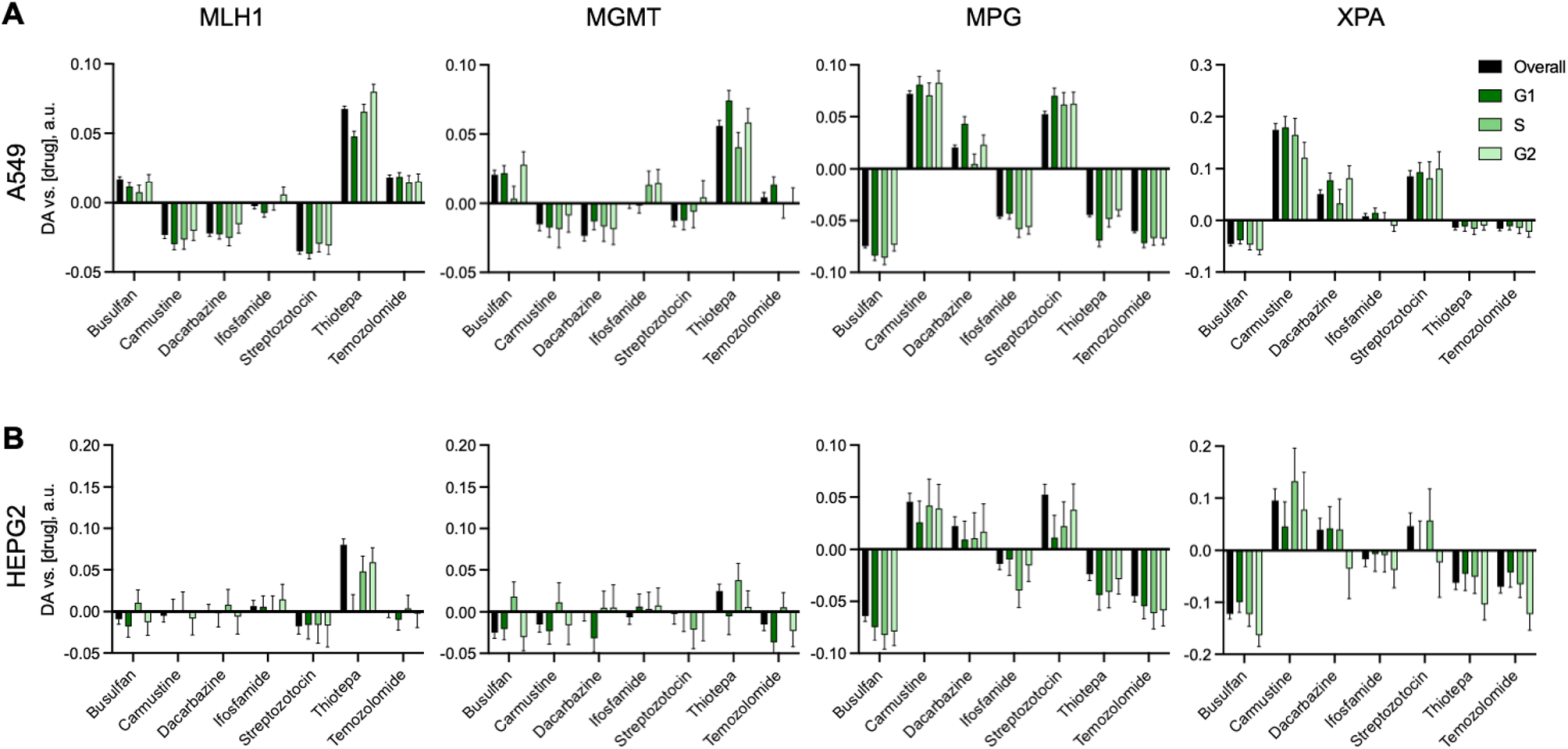
Cell cycle impact on DNA damage response protein recruitment induced by alkylating agents. (**A** and** B**) Autocorrelation DA versus concentration linear regression slope for all cells (overall) and cells in each of the cell cycles for each of the alkylating agents in A549 (A) and Hep2 (B) cells. Shown are linear regression slope with standard error of the fit.

Because we are imaging with immunofluorescence it is simple to label another protein and evaluate recruitment to DNA alkylating agent induced DNA damage. Therefore, we sought to measure recruitment of proteins known to be responsive to alkylating agents: specifically, the mismatch repair protein (MMR) MLH1, MGMT, which removes O_6_MeG lesions, MPG, which is a base excision repair protein, and XPA, which is an excision repair protein. Based on recruitment patterns of the four proteins, the alkylating agents could be separated into 2 distinct groups (Fig. 5). Streptozotocin, carmustine and dacarbazine reduced clustering of MLH1 and MGMT but induced clustering of MPG and XPA. While busulfan, thiotepa and temozolomide all reduced clustering of MPG and XPA while inducing clustering of MLH1 in A549 cells and showing mixed results in MGMT response. Ifosfamide reduced MPG recruitment but did not impact any other proteins. Protein response to alkylating agents was independent of cell cycle (Fig. 6). Strikingly, MGMT is involved in the removal of O_6_MeG lesions and MLH1 is a major protein in MMR, which occurs when O_6_MeG lesions are not removed^30^, whereas MPG and XPA are both involved in excision repair^31,32^. Since these proteins cluster by repair mechanism it appears that the alkylating agents induce two distinct repair pathways. Furthermore, streptozotocin, dacarbazine and carmustine are all in the nitrosourea class of alkylating agents that alkylate through an S_N_1 mechanism^33,34^. However, busulfan acts through an S_N_2 mechanism^35^ while thiotepa and temozolomide need to be first activated^36,37^ before alkylating in an S_N_2 fashion. Thus, these results suggest that the mechanism of alkylation dictates the repair pathway and protein recruitment pattern. These results also demonstrate that autocorrelation analysis can be carried out at different protein expression levels and intensities in the imaging results (Fig. 5A). Thiotepa induced a significant response in all four proteins, however there is no resolvable impact of thiotepa on the distribution of these proteins within the nucleus (Fig. 5A). Thus, autocorrelation analysis is essential to evaluating response of DDR proteins in the DNA damage pathway.

## Discussion

Phenotypic screening is a robust tool to study the impact of molecules on cellular function. However, morphological screening typically evaluates cellular shape through organelle specific labels. Therefore, existing approaches may overlook specific protein responses that are indicative of cellular pathways. In the original analysis of the 1,008 compound imaging dataset no DNA alkylating agent compounds were found to be active. Furthermore, in A549 and WPMY-1 cell lines, the use of TP53BP1 as a marker actually reduced the ability to detect PARP inhibitor activity compared to other protein markers. This was similar for other MoAs that act on DNA or the DNA damage response, such as bromodomain inhibitors and HDAC inhibitors. Considering that 53BP1 is heavily involved in the DNA damage response pathway^38,39^ and many MoAs of the compounds screened act to interfere with the DNA damage response, alter the cell cycle or impact the amount of DNA damage in a cell, it was surprising that 53BP1 was not a more sensitive marker in the phenotypic screen. Traditionally, the recruitment of 53BP1 to sites of DNA damage is only resolved through pre-extraction and immunofluorescence^40^. In this process, labile nuclear 53BP1 protein is extracted from the cell prior to fixation to reduce the background concentration and increase the resolution of chromatin-interacting 53BP1 in DNA damage foci. The phenotypic screen analyzed here used live cells with fluorescently labeled, endogenous 53BP1, which prevents removal of protein not interacting with DNA and reduces the ability to resolve 53BP1 foci. This limitation likely prevents traditional phenotypic analysis from detecting non-resolvable spatial signaling of DNA damage response proteins. Unfortunately, pre-extraction is a subjective process that potentially removes protein associated with chromatin and DNA and thus not a robust approach for phenotypic screening generally^41,42^.

Applying spatial image autocorrelation overcomes the limitation of high non-foci background fluorescence to quantify the degree of 53BP1 protein clustering within the nucleus. Here, we found autocorrelation analysis is able to detect activity of compounds that generated no measured activity when analyzed by traditional phenotypic analysis. These results confirm that many of the compounds associated with DNA damage, DNA damage response or cell cycle indeed have activity in the cell lines used in the screen. Optimizing imaging by collecting images without pixel binning greatly enhanced the sensitivity of autocorrelation to detect DNA damage response activity. To achieve sensitive autocorrelation analysis the pixel/voxel size needs to be lower than the point spread function (beam width). In imaging experiments from our laboratory, which did not bin pixels, we measured significant 53BP1 response to nearly all alkylating compounds in two cell lines. Pixel binning is routinely performed in phenotypic screens to reduce the amount of data, however there is no other advantage and thus imaging without binning is amenable to any experiment. Presumably binning does not impact sensitivity of phenotypic screens, however we found that autocorrelation analysis was significantly enhanced in the absence of binning. Autocorrelation measures changes in spatial intensity fluctuation that can be impacted my any biological phenomenon that changes the distribution of the protein of interest. However, the shape of the region analyzed should not impact the results.

Expanding measurements to proteins specific for different pathways of the DNA damage response demonstrated that autocorrelation analysis was able to classify alkylating agents and the DNA damage response by mechanism. This remarkable sensitivity from simple immunofluorescence imaging suggests that autocorrelation analysis is a much more robust tool than phenotypic screening when screens are intended to be more focused. Autocorrelation could serve as a unique resource to identify compounds that target the DNA damage response or study mechanisms of resistance to DNA targeting drugs. Overall, these results suggest that more complex analysis of specific, yet broadly functional fluorescent labels can reveal compound activity that is not otherwise detectable.

## Methods

### Analysis

Images from the original study^15^ were accessed through the Image Data Resource^43^ API on the Open Microscopy environment. Nuclei were segmented using StarDist^44^ on the BFP channel image. Segmented nuclei were then analyzed by image correlation spectroscopy autocorrelation^45^ in the TP53BP1 channel and the average degree of aggregation was calculated. Briefly, a 2D Fourier transform of segmented nuclei was performed in SciPy and multiplied by the complex conjugate. The inverse Fourier transform was taken and a 3D curve with the equation y = a*exp((− (x − x_0_)^2^ + (y − y_0_)^2^)/c^2^) + b was fit to the real values of the results to determine the correlation peak. Here x_0_ and y_0_ are set to the peak of the autocorrelation curve and height of the curve (a) represents the inverse average number of independent particles per point spread function (or beam width). The degree of aggregation (DA) was calculated by the dividing the average nuclear intensity by the number of particles per point spread function. We have deposited the Python scripts used to perform these calculations on our GitHub page: github.com/dubachLab. For each cell line, compound induced DA was plotted against the compound concentration and linear regression was performed with a linear model in R using the map function. Fitting was performed on the log of the concentration with DA values averaged with standard deviation for each concentration. DMSO control was included in the fitting and the concentration set to 3 orders of magnitude lower than the lowest dose used. MoA activity was determined for MoAs with at least 5 compounds with linear regression results in each of the cell lines. Active compounds were defined as having significant (*p* < 0.05) non-zero linear regressions, either negative or positive. Correction using Benjamini–Hochberg FDR adjustment of p-values was also performed, however to directly compare results to the original screen analysis we did not apply this to the fit data, but did include results in Table S1. The activity score was determined by summing the direction of each active compound and dividing by the total number of compounds in the MoA.

Image access and autocorrelation analysis was performed in Python, while results analysis and plotting were performed in R and Prism.

### Cell culture

HepG2 and A549 were obtained from ATCC and cultured in RPMI with 10% FBS and 1% pen/strep. Cells were plated into glass bottom 384 well plates (Cellvis) and allowed to adhere overnight. DNA alkylating agents (Caymen) were added in DMSO at the desired concentration in fresh media and the cells were incubated overnight. DMSO was maintained at a constant concentration in each well.

### Immunofluorescence

After DNA alkylating agent treatment for 24 h cells were fixed with 16% PFA (EMS) freshly diluted to 4% in PBS (Thermo) for 15 min at room temperature. Plates were washed 3 times with PBS and cells were blocked for 1 h at room temperature on a rocker in blocking buffer (5% normal goat serum (CST) and 0.3% triton-X (Sigma) in PBS). Cells were then incubated with primary antibody (1:500) in antibody dilution buffer (1% BSA (Tocris) and 0.3% triton-X in PBS) overnight at 4 ºC on a rocker. Cells were then washed 3 times in PBS and incubated with fluorescent secondary antibody at 1:1000 in antibody dilution buffer for 1 h at room temperature on a rocker. Cells were washed 3 times in PBS and labeled with DAPI (Thermo) at 1 μg/ml for 5 min. Cells were washed 2 times in PBS and transferred to the microscope. Images were taken on a Nikon Ti2 widefield microscope in DAPI, Cy3 and Cy5 channels using standard filter cubes and a 20x NA 0.75 air objective.

### Antibodies

**Table:** 

53BP1	Cell Signaling Technologies	#88439
MLH1	Cell Signaling Technologies	#3515
MGMT	Cell Signaling Technologies	#58121
MPG	Thermo Fisher	#MA5-19353
XPA	Thermo Fisher	#PA5-84315
Anti-rabbit IgG	Cell Signaling Technologies	#4414
Anti-mouse IgG	Cell Signaling Technologies	#4409

### Supplementary Information

Supplementary Table S1.

## Data Availability

All data generated or analyzed during this study are included in this published article and supplementary file. Raw microscopy images are available from the corresponding author on reasonable request.
